# Everyday life and Everyday Leisure

**DOI:** 10.1007/s41978-023-00134-0

**Published:** 2023-04-04

**Authors:** A.J Veal

**Affiliations:** 1 Business School, University of Technology Sydney, Sydney, Australia; 2 Broadway, NSW 2007 PO Box 123, Australia

## Abstract

A substantial body of theory exists on the concept of everyday life, including the sociology of everyday life, but it has barely featured in the mainstream of the sociological study of leisure or leisure studies more broadly. This paper explores this theoretical work and considers the place of leisure in it, and how it might inform the further development of the study of leisure. It is argued that the time is right to broaden the scope of leisure research to incorporate consideration of the way in which all forms of everyday time-use interact.

## Introduction

The exploration described in this article began with the observation that a number of prominent strands of leisure theory are focussed on elevated or ‘special’ forms of leisure. These include: ‘serious leisure’ (Stebbins, 2007); ‘devotional leisure’ (Blackshaw, 2017); Aristotelian leisure based on the Greek *scholê* (De Grazia, 1962; Van Moorst, 1982; Kalimtzis, 2017) and leisure as a state of ‘flow’ (Elkington, 2017) or self-actualization (Dumazedier, 1974; Csikszentmihalyi & Kleiber, 1991). These constructs seem to reflect Huizinga’s (1950/1955, p.28) declaration that leisure is simply ‘different’ from ‘ordinary life’. For most people, however, most of their leisure time and activity can be described as *everyday*. It therefore forms part of *everyday life*, along with other everyday activities, including most (paid and unpaid) work, family and social life and self-maintenance activity. The concept of everyday life as a whole has, nevertheless, not featured prominently or explicitly in the study of leisure. Much leisure research activity is focussed on the rare and exceptional rather than the typical, thereby ignoring the bulk of leisure experience.

Beyond leisure research, however, everyday life has been recognised as significant by a myriad of social theorists, as indicated in overviews by Bennett and Watson (2002), Highmore (2002a) and Gardiner (2000). It is a major focus of anthropology and there is also a distinct *sociology of everyday life*, as indicated in reviews by Adler, Adler and Fontana (1987), Kalekin-Fishman (2013) and Sheringham (2006). It has even been referred to as a ‘third sociology’ (Sztompka, 2008).

Research on the sociology of everyday life has, nevertheless, featured from time to time in and around studies of leisure:

- Well-known to leisure scholars is Goffman’s (1959) *The presentation of self in everyday life*, involving analysis of routine inter-personal interactions using dramaturgical metaphors.
- The collection of data on patterns of daily activity in the form of time-use surveys had its origins in the early part of the twentieth century (Sorokin & Berger, 1939; Robinson et al., 1972; Pentland & Harvey, 1999, p.5) and continues to this day (e.g., Gershuny & Sullivan 2019).
- Also familiar to many is Cohen and Taylor’s (1976/1992) *Escape attempts: The theory and practice of resistance to everyday life*, in which the main theme is the notion of escaping from the grip of the mundane everyday, typically via forms of leisure activity.
- The study of leisure has been substantially influenced by cultural studies, which includes a concern for the everyday. Chaney (2002, p.12), for example, observed that ‘everyday life has become the dominant theme and idiom of cultural life in later modernity’.
- While the concept of *flow* is associated with exceptional, non-everyday types of leisure and work experience, the related concept of *microflow* refers to the routine patterns of the everyday (Csikszentmihalyi, 1975, pp.140–160; 1997; Hektner et al., 2007).

While the idea of the everyday has largely been neglected in mainstream leisure research, this has not been total. A special issue of *Society and Leisure* devoted to ‘Leisure in the context of everyday life’ (Zuzanek & Larsen, 1993) included a number of articles typically focussed on descriptive studies of daily and weekly schedules of leisure, although with little reference to the theoretical sociology of everyday life. Furthermore, there have been exhortations for leisure scholars to move away from studying leisure activity in isolation from other aspects of daily life. For example, 35 years ago, Mommaas and van der Poel (1987, p.164) observed a ‘growing awareness of the fact that the way people spend their free time cannot be studied isolated from the other practices they participate in daily’. They also cited as a way forward (p.171) the work of de Certeau (1980) on the everyday (see below). More recently, Kenneth Roberts (2006a, b) has argued that the most powerful explanations of leisure behaviour are those that ‘take account of the actors’ total situations and the broader ways of life or lifestyles to which specific uses of leisure contribute’ (p.225).

Against this background, there has to date been no attempt to develop the link between the sociology of leisure and the sociology of everyday life. The aim of this paper is to fill this gap. It identifies a rich vein of theory and empirical research on everyday life which has the potential to enhance the study of leisure in productive ways.

The paper is not in the form of a systematic review of the literature; it is an informal exploration. It began with Stebbins’ *Pondering Everyday Life* (2020), which was identified while conducting research on the concept of serious leisure (Veal, 2021). In this volume Stebbins highlights the idea of *everyday activities* in the context of leisure, work and domestic obligations, but devotes less than half a page to examining the related sociological research on everyday life (p.17). Nevertheless, it provided a valuable starting point, with one source leading to another, in a ‘snowball’ type of process, while existing overviews (Adler et al., 1987; Kalekin-Fishman, 2013; Sheringham, 2006) were also helpful.

The paper is in four sections: first, a brief historical discussion of the contextualisation and definition of the study of everyday life; second, an overview of largely sociological theoretical perspectives on the everyday and a brief examination of what they have to say about leisure; third, a consideration of the extent to which, despite the apparent general neglect, some researchers in leisure and related domains have addressed the concept of everyday life; and a discussion of further implications for the study of leisure.

### Contextualising and Defining Everyday life

Adler et al. (1987) provide an account of the development of the sociology of everyday life as an application, mostly in America, of symbolic interactionism, phenomenology and ethno-methodology associated with the likes of Schutz, Garfinkel and Goffman. This tradition emphasises the micro-level of analysis with little or no attention given to macro-level, structural, society-level issues. A more recent review by Kelekin-Fishman (2013), however, locates the origins of the study of everyday life in 1950s France and a small group of Marxist social theorists, the Situationists (Kaplan & Ross, 1987), who were associated with Henri Lefebvre, the ‘quintessential critical theorist of everyday life’ (Gardiner, 2000, p.71). An account by Schilling (2003) also focusses on 1950s French origins, while Sheringham (2006, p.3) observes that this early body of work placed ‘the question of the everyday at the heart of French culture in the last two decades of the twentieth century and into the new millennium’. Eley (1995) refers to a similar, although less prominent, development of interest in everyday life, or *Alltag*, among German historians, from the 1970s. Roberts (1999, 2006a, b) takes the historical account of the study of the everyday back to 1917 and the Russian Revolution, arguing that its recent treatment, at least among contemporary cultural studies commentators, has ‘suffered not only from a general process of critical dehistoricization but an acute philosophical foreshortening’ (2006, p.2). This was partly due to the often considerable delays in publication of French and German works in English translation.

Featherstone (1992) argued, from a cultural studies perspective, that ‘everyday life is the life-world which provides the ultimate ground from which spring all our conceptualizations, definitions and narratives’ (p.160). He suggested five defining features of the concept:

1. ‘What happens every day, the routine, repetitive taken-for-granted experiences, beliefs and practices; the mundane ordinary world, untouched by great events and the extraordinary’.
2. ‘The sphere of reproduction and maintenance, a pre-institutional zone in which the basic activities which sustain other worlds are performed, largely by women’.
3. An emphasis on the present, ‘which provides a non-reflexive sense of immersion in the immediacy of current experiences and activities’.
4. An emphasis on ‘common sensuality, being with others in frivolous, playful sociability’.
5. An emphasis on ‘heterogeneous knowledge’ in which ‘speech and “the magic world of voices” are valued over the linearity of writing’ (Featherstone, 1992, pp.160–161).^1^

The first of these features involves three dimensions: the *temporal*, indicating that it happens ‘every day’ and is ‘repetitive’, exemplified in the leisure context by daily television-watching (Fisher & Robinson, 2010; Gershuny & Sullivan, 2019); the *cognitive/normative*, viewing the everyday as routine, taken-for-granted and mundane; and the *relative*, referring to the everyday as being ‘untouched by great events and the extraordinary’.

Featherstone’s second feature, concerning reproduction and maintenance, echoes Joffre Dumazedier’s (1967, pp.16–17; Veal 2019a) well-known functional definition of leisure, with its first two functions being the everyday activities of *rest* and *diversionary entertainment*, seen as facilitating recuperation from work. This reference to work highlights Featherstone’s apparent exclusion of work from his definition, although the reference to reproductive and maintenance activity clearly includes unpaid work. Other commentators have generally included paid work in the everyday, including Lefebvre (1958/2014, p.53), Heller (1970/1984, p.60) and Felski (2000, p.16). Baudrillard (1970/1988, p.35) defined ‘everydayness’ as a realm in which the individual seeks to organize ‘work, leisure, family, acquaintances … in a coherent system’.

Featherstone’s third definitional feature indicates a focus on the present. This could be referring to what Maffesoli (1989b, p.2) terms ‘presentism’, that is, concentrating on the lived life without considering the prospect of an after-life or hoped-for collective future utopia: in Felski’s (2000, p.16) terms: ‘a world leached of transcendence’. However, it may also imply a more limited preoccupation with the present with little or no thought for the past and future within this life, which is surely challengeable. As Hilbrecht (2013, p.178) puts it: ‘While going about daily activities, people live in the present but may be focused on past experiences or future commitments’.

The fourth feature identifies the everyday with ‘frivolous, playful sociability’. However, boring and even painful experience must also be involved, as suggested by the use of the term ‘mundane’ in the second feature and in the work of Cohen and Taylor (1976/1992, p.46), noted above.

Featherstone’s fifth feature, concerning ‘heterogeneous knowledge’, posits that the information and skills required to successfully negotiate everyday living are acquired from numerous sources, including the informal, such as received ‘common-sense’.

An inclusive conception of everyday life guides the discussion which follows, with a key emerging implication being that, if leisure is to be understood in terms of patterns of everyday behaviour, then all the other categories of everyday activity which compete for an individual’s time and attention must be part of the understanding.

### Theorising Everyday life

This section of the paper comprises five sub-sections: micro and macro levels of analysis; Marxist theorists; post-Marxist perspectives; cultural studies; and a *s*ummary and critique.

#### Micro and Macro Levels of Analysis

Some analyses of everyday life can be seen as a field of *microsociology* (Collins, 1981), which contrasts with the macro-level of analysis, involving the state, organizations, class, the economy and culture. Thus, pioneering proponents of the micro-level of sociological analysis, Berger and Luckman (1972), while critical of the then state of macro-level sociological theory (p.208), showed a reluctance to engage with it themselves. As Felski (2000, p.27) put it: ‘phenomenological studies of everyday life are concerned with description rather than explanation and thus do not address questions of politics and power’. Furthermore, Chaney (2002, p.42) notes the lack of a ‘sense of historical change’ in ethnomethodology, while Kenneth Roberts (2016, p.116) notes the failure to ‘build upwards from micro-studies to meet the classical grand theories’. However, Norbert Elias, in his essay, ‘On the concept of everyday life’ (1978/1998), while noting the divide between the work of ethno-methodological and phenomenological sociologists and that of neo-Marxist theorists, argued that there was no good reason why there should be any incompatibility between investigation of macro-level ‘structures of social life’ and of the micro-level ‘meanings of aspects of social life as experienced by the people’ (p.168). Thus research perspectives can encompass both micro and macro levels of analysis (see Alexander, Gieson, Mȕnch & Smelser, 1987). In this paper, therefore, the focus is on such inclusive perspectives.

#### Marxist Theorists

A common response of critical theorists of the 1950s and 1960s to the fact that capitalism had not brought about the material immiseration of the masses anticipated by Marx, was to argue that a general state of *false consciousness* or *false needs* had been superimposed upon individuals by social interests involved in their repression (Marcuse, 1968, p.21). While this view can be seen as making challengeable assumptions about the passivity and lack of agency of individuals as consumers and citizens, it also implied that some research attention should be focussed on the everyday life of the workers/masses in which repression was being experienced. This would involve an adaptation of the traditional Marxist perspective.

One adapter was Henri Lefebvre who, in his classic study, *Critique of Everyday Life*, observed that, from as far back as ancient Greece, the everyday life of the masses had been deprecated by intellectual, economic, political and cultural élites (Lefebvre, 1958/2014, p.51), whose own lives, by contrast, involved engagement with philosophy and other intellectual pursuits and with the arts and government (p.108). However, he argued, a civilization was defined by the quality of everyday life experienced by the masses and this was also the site of dissatisfactions which could have the potential to spark demand for change, even revolution (p.109). Therefore, Lefebvre argued, Marxist theory should be informed by and engaged with these everyday lives. Furthermore, he added that sociology should ‘study the way of life of workers as such, their place in the division of labour and in the social system’ which was in part ‘reflected in leisure activities, or at least in what they demand of leisure’ (p.52). There was, however, ‘a certain obscurity in the very concept of *everyday life*’. Was it to be found in work, in leisure or in family life? The answer was, it involved all three elements (Lefebvre, 1958/2014, p.53). He noted that capitalist society sought to divide these components into separate functional spheres. The leisure sphere had been shaped by market forces for relaxation and for the consumption of diversionary entertainments. The latter were designed as relief from the everyday realm, which was viewed negatively as being associated with ‘worry and necessity’, and with being ‘tired and tense’ and ‘anxious, worried and preoccupied’ (p.55).

In promoting such a focus, however, Lefebvre came into conflict with fellow Marxists, who believed that the trigger for revolution remained direct conflict between labour and capital in the industrial sphere. This disagreement, and his rejection of Stalinism, eventually led to his expulsion from the French Communist Party.

The first edition of volume I of *Critique* had been published in 1947, following the Liberation of France, when there was an atmosphere of optimism among the political left concerning the possibility of a changed post-war society. In his later volume, *Everyday Life in the Modern World* (1971/2016), Lefebvre indicated that the optimism of 25 years earlier had been misplaced. Far from being overthrown by revolution, capitalism had increased its grip, becoming the *bureaucratic society of controlled consumption*. Everyday life was increasingly dominated by a closed system, in which consumer demands were manipulated by capital (p.65). While this comes close to the ‘false consciousness’ argument, the circuit was ‘not completely closed’ because people still retained certain ‘irreducible’ levels of agency. The only way to stop the circuit from closing completely, however, was to ‘conquer the quotidian, attack it and transform it’. Intellectuals might have been expected to play this role but their limited specialist perspectives had obscured the everyday as a distinct and significant phenomenon. While Lefebvre continued to believe in the potential to act politically to bring about change (p.68), the focus for change/revolution had shifted: everyday life had ‘taken the place of economics’ and should be the focus of ‘a *cultural revolution* with economic and political implications’ (p.167, emphasis added). This line of reasoning resulted in a political agenda, albeit one developed along the lines he had originally been critical of, namely a prescription arising from an élite intellectual source. He declared: ‘The theoretical revolution which constitutes the first step towards a cultural revolution is based on philosophical experience’ (Lefebvre, 1971/2016, p.173). The cultural revolution included sexual reform, the festival ‘rediscovered’ (pp.174–175) and urban reform (see Gottdiener 1993).

Marxist theorist Agnes Heller (1929–2019) contributed significantly to the theory of everyday life, initially in Hungary in the 1970s (Heller, 1970/1984) and later in Australia and the USA (Heller, 1987). Like Lefebvre, she was expelled from the communist party for her unorthodox views (Gardiner, 2000, p.127). Heller’s schema of society involved three *spheres*, referred to using the unwieldy Marxian term *objectivations*.

- The *objectivation in itself* sphere is everyday life, which involves rules, norms and practices related to meanings, language and interactions with fellow humans and the material world, to which the individual is exposed from birth and takes largely for granted.
- The *objectivation for itself* sphere involves philosophy, religion etc., where meaning is provided.
- The *objectivation for and in itself* sphere comprises one or more socio-economic-political institutions, the growth of which characterises modernity (Heller, 1987).

Heller saw the third sphere becoming so powerful that the everyday *objectivation in itself* sphere disappeared or ceased to function effectively. As in Huxley’s ‘brave new world’ (p.310), ‘the human condition vanishes’ (p.313). This corresponded to Habermas’s ‘colonization of everyday life’ by the forces of ‘instrumental reason’ (discussed below), which she acknowledged.

Canadian Marxist-feminist Dorothy E. Smith argued, in *The Everyday World as Problematic* (1987), that the discipline of sociology had created a discourse which ignored the unpaid and paid work of women. She posited that a feminist sociology should begin by examining the experience of everyday life, particularly that of women. While initially drawing on the micro-level work of Schutz, she saw the need for a macro-level dimension to the analysis. She declared: ‘an inquiry confining itself to the everyday world of direct experience is not adequate to explicate its social organization’ (p.89), since the everyday world was continually impacted by forces beyond it. While she clearly accepted a Marxist perspective on the workings of those macro-level forces (p.95), Doran (1993) argues that this can be seen as involving an imposition of an external ideological frame, the very approach she was seeking to avoid (Smith, 1987, p.107).

While the commitment to a Marxist perspective was generally abandoned by subsequent theorists, it remained theoretically influential, partly because it demonstrated that the micro-level concept of everyday life could be studied in the context of critical macro-level social theory.

#### Post-Marxist Perspectives

British sociologists Stanley Cohen and Laurie Taylor (1976/1992, p.231) explicitly deprecated (unidentified) Marxist theorists who attempted to ‘reconstitute marxism as a critique of everyday life’ while contemptuously ignoring people’s ‘everyday struggles …by reference to the false consciousness of the masses’. They argued that the masses were not receptive to such messages since they would ‘surely not draw much consolation about the poverty, alienation and boredom of their everyday life from being told that it needs a new praxis for its transformation’ (p.233). Cohen and Taylor’s pragmatic approach nevertheless portrayed everyday life under capitalism as akin to an ‘open prison’, referring to it as *paramount reality*, after Berger and Luckman (1972). In this setting individuals negotiated their social and economic position and roles and developed identities in the context of lives of routine and an ‘awful sense of monotony’ (p.46). This involved *identity work* comprising ‘ingenious, complex and even desperate’ resistance and escape manoeuvres to evade the clutches of paramount reality (p.43). These were: ‘free areas, escape routes and identity sites’ (p.112) which took the form of: *activity enclaves* (hobbies, games, gambling, sexual activity); *new landscapes* (holidays, mass culture, art); *mindscaping* (drug-taking, therapy); and joining *alternative communities.* However, these ‘escapes’ did not necessarily operate as expected because they could be merely a means of adapting to, rather than changing, paramount reality. They were themselves often commodified and therefore part of the paramount reality itself, rendering it tolerable rather than acting as an escape from it (p.154).

Considering the macro-sociological implications, Cohen and Taylor rejected the revolutionary prescriptions of early critical theorists, such as Marcuse and the Surrealists (p.231), arguing that bringing about a transformation of consciousness sufficient to fundamentally transform the everyday life of the masses was unlikely (p.233). They found ‘more consoling’ the idea of individual action in pursuing escape strategies to achieve social change: ‘I escape therefore I am’ (p.236). However, they admitted that this could be seen as ‘bourgeois individualism’ and ‘limited as an ideology’. So the result was uncertain in that neither the traditional revolution scenario or aggregate individual periodic escape activity offered a realistic approach to a changed society. The cul-de-sac did not, however, end there. A second edition of the book included the original text unchanged but with the addition of a lengthy new introduction (Cohen and Taylor, 1976/1993, pp.1–29). In this, they reflected on how their conclusions might have been changed in light of the insights offered by postmodern thinking over the intervening 15 years. They declared that they would have made a ‘massive narrative change’ to shift their emphasis from ‘the subversion or deconstruction of escape attempts’ to the ‘deconstruction of paramount reality’ (p.3). The paramount reality from which people sought to escape was no more solid than the escape routes and identity sites to which they escaped.

Pierre Bourdieu’s (1979/1984) analysis of social structure challenged the above frameworks with their view of an undifferentiated working class living everyday lives and a bourgeoisie controlling the conditions of such lives. Empirically, he presented some 26 *class fractions* of French society, ranging from unskilled workers to ‘commercial employers’ and ‘industrialists’. These were located in two overlaid conceptual spaces: the ‘space of social position’ and the ‘space of lifestyle’, structured by possession of economic capital (income and occupation) and cultural capital (formal and informal education and socialisation) (pp.128–129). Each class fraction had a distinctive *habitus* reflected in a lifestyle which comprised: ‘all the properties … with which individuals and groups surround themselves – houses, furniture, paintings, books, cars, spirits, cigarettes, perfume, clothes – and the practices in which they manifest their distinction – sports, games, entertainments’ (p.173). This list clearly encompassed everyday accoutrements and activities. It omitted paid and unpaid work activity and did not refer explicitly to family activity, but they were seen as part of a society-wide ‘system of classified and classifying practices’, so it can be said that the social structure was inextricably entwined with the everyday. The context for social change lay not in an economic struggle between just two classes but in a competitive process among class fractions in which each ‘strives to maintain or change its position in the social structure’ (p.157) comprising cultural – and everyday – phenomena. However, the result, even if all groups improved their absolute conditions, was invariably the reproduction of the structure and its relativities (p.165). The only chance of this pattern being disrupted was some sort of economic crisis (p.168).

Bourdieu’s concepts of *habitus* and the related culture/consumption-structured social system have been subject to some criticism. First, it has been found empirically that, while the location of class fractions in the spaces of social position and lifestyle is based on a sort of centroid for each of the various lifestyle/consumption variables, often only a minority of members of the class fraction are located at or even near the centroid (see, for example: Bennett, 2007, p.216; Bennett et al., 2009, Fig. 3.9). The second, related, criticism is that the concept of *habitus* exaggerates the homology among the practices, or lifestyles, within individual class fractions. Lahire (2003) argues that, rather than being boxed-in to one *habitus*, the individual is a plural being, with a unique constellation of dispositions based on personal history as well as class context. Such arguments are supported by the idea of *cultural omnivores* (Chan, 2010), who do not conform to the predicted habitus-class homology. Add to this the massive increase in the variety and capacity of communications media in the half century since Bourdieu’s empirical data were collected and it is likely that his schema is at least out of date in its particulars. This is confirmed in the somewhat frustrated attempt by Bennett at al. (2009) to replicate the study using data from twenty-first century England. Despite criticism, however, Bourdieu established that lifestyle/everyday life, with its mass of relevant variables, could be studied empirically and theoretically, both quantitatively and qualitatively, and at both micro and macro levels.

**Fig. 1:**
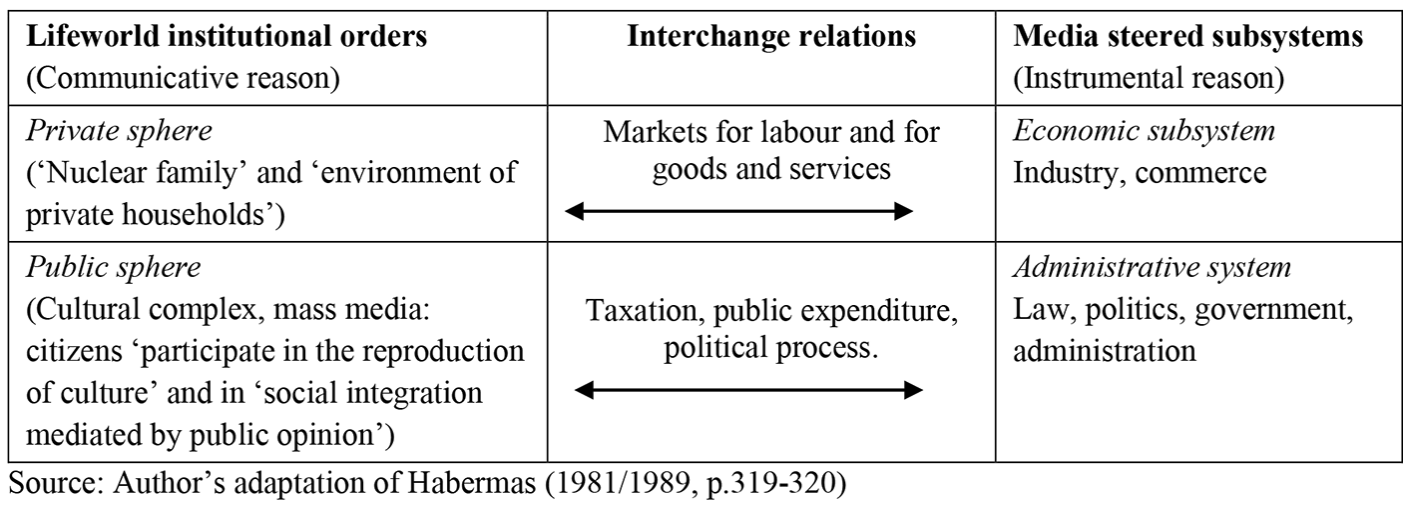
The Habermas schema: the lifeworld and system/steering media

Jȕrgen Habermas focused attention on the concept of *lifeworld*, developed from the phenomenological work of Husserl and Schutz (Habermas, 1981/1989, pp.113–197). In his theoretical framework of society, the *lifeworld* sphere comes into play at the micro-level when individuals, workers and social groups interact, involving the largely taken-for-granted or sub-conscious medium of *communicative reason* which characterises free social intercourse. A second sphere of activity and communication in the schema is the *system* or *steering media* of large-scale markets, finance, government and the law, which is guided by *instrumental reason* associated with phenomena such as the profit motive and the exercise of power. In very simple societies, the two spheres coincide, involving the same relatively small number of individuals. As societies become more complex, the size and number of instrumental institutions grow and the expanded steering media system becomes separated from the lifeworld of the masses. The lifeworld and steering media spheres are each divided into two, with various relationships between the subsections, as summarised in Fig. 1.

Using the lens of the Habermas schema, recent decades have seen a tendency for the *instrumental reason* system to seek to increasingly ‘colonize’ the lifeworld of the masses by, for example, commodifying and marketizing or bureaucratising activities which had hitherto been exclusively part of the communicative/everyday lifeworld. This colonization process is resisted by those living in the lifeworld, which is concerned with ‘cultural transmission, social integration, and child rearing, and remains dependent on mutual understanding as a mechanism for coordinating action’ (p.330). The Habermas thesis implies that the colonization process suppresses these communicative functions as features of the lifeworld/everyday. The extent to which this process disrupts the communicative characteristics of the private sphere is at least partly an empirical question, but it has been argued that Habermas ‘failed to develop a sustained empirical program at the level of the everyday’ (Doran, 1993, p.6). Nor did he prescribe a solution to the political action problem identified, that is, what is to be done about it.

Arguably, the colonization process breaks down the boundaries between the public and private spheres. This might be particularly evident in leisure contexts, for example, family life in the context of broadcast and streamed mass media and friendship in the context of, for example, the corporately-owned pub or ‘family restaurant’. While everyday life can be seen as anchored in the private sphere, leisure scholars drawing on Habermas’s work have tended to focus on the public sphere, possibly prompted by Habermas (1962/1992) having devoted a whole volume to the topic. For example, Kenneth Roberts (2016, p.101) suggests that a key question for leisure scholars arising from Habermas’s work is ‘whether the present-day public spheres they study can become sites of more widespread emancipation from the instrumentalities that dominate economic life and politics’. Some twenty years earlier, Hemingway (1996) had expressed confidence that they could, and that such emancipation was the key task of leisure studies. The leisure scholar most clearly identified with Habermas, Karl Spracklen, while similarly concentrating on the public sphere (2009, pp.72–80), adopts a more active tone: ‘We need to fight to protect communicative leisure, activities that are local, democratic, leaderless, anarchic, private and free’ (Spracklen, 2013, p.238). However, he also recognises the possibility of private sphere everyday leisure activity surviving the colonization process, illustrated by case studies of friendship in pubs and dancing in nightclubs (pp.235–237).

Michel Maffesoli’s most well-known work, *The Time of the Tribes* (1988/1996), has typically been cited by leisure researchers as a contribution to discourse on lifestyles (e.g., Rojek 1995; Spracklen, 2009, p.123; Veal 2013). However, the *neo-tribe* concept is part of a wider framework centred on the ‘humdrum, the normal and the everyday’ and, furthermore, providing ‘a solid basis for the whole of sociology’ (Maffesoli, 1985/1996, p.136). Reflecting Lefebvre’s strictures against intellectual élites imposing social theory on the masses, Maffesoli declared that intellectuals had a tendency to ‘approach a subject *in absentia*’ and investigate and then present their diagnoses, indicating ‘a certain inborn mistrust of the common sense of the masses’ (1988/1996, p.56), largely due to the latter being deemed to be ‘shamelessly preoccupied … with the materiality of life’. Social scientists had sought to mould the masses into their own conception of a ‘subject of history’, while still perceiving them in a ‘pejorative sense’ (p.57). Maffesoli (1989a) argued that, rather than engaging with the ‘exhausted’ rationalities of the ‘great systems of interpretation such as Marxism or Freudianism’, social research should seek to discover and understand the ‘mixture of feelings, passions, images and differences’ to be found in everyday life.

Also reflecting Lefebvre, Maffesoli was of the view that ‘history and significant political events are above all the creation of the masses’ (1988/1996, p.58, see also, 1989b, p.4). He offered the French term *puissance* (collective life-force, or will to survive) as the key ‘being-together’ characteristic of the masses – a secular equivalent of the convivial quality of religion (p.58). The existence of *puissance* could give rise to particular groupings – or *neo-tribes* – extending from a small local group to a whole nation and beyond. Any one individual might identify with a number of groupings/tribes. In the closing decades of the twentieth century, Maffesoli saw a decline in individualism and an increase in mass identities: ‘The conformism of youth, the passion for likeness within groups or ‘tribes’, the phenomena of fashion, standardized culture, up to and including the unisexualization of appearance, permit us to claim that what we are witnessing is the loss of the idea of the individual in favour of a much less distinct mass’ (Maffesoli, 1988/1996, p.64). This contrasts with a number of contemporary theorists, such as Giddens (1991, pp.5,80–81) and Beck (1992, p.88), who have observed a tendency *towards* individualism. However, Sweetman (2004) has argued that the two tendencies can work in tandem, giving the example of people with tattoos seeing the tattoo as both an individualist statement and a claim of affinity with fellow tattooees making similar individualist statements.

Recent political events have thrown up numerous examples of large Maffesolian neo-tribes on the political scene, including: the Black Lives Matter movement in the USA and beyond; the *gilets jaunes* in France; and the global #MeToo movement. Examples related to the everyday and leisure include: supporters of sports teams, particularly non-registered supporters; ‘trekkies’ who are devotees of the television series *Star Trek*; ‘stanning’ fan groups; and groups focussed on certain reality TV shows, music genres and even commercial products (see Cova, Kozinets & Shankar, 2007). Maffesoli was writing before the advent of various social media, which now clearly facilitate the rapid growth of such neo-tribes. The key feature of tribal groups is that they are, for the most part, informally organised and marginal to the structures of mainstream society, although some develop a certain level of institutionalisation over time. Furthermore, in regard to the focus of this paper, they tend to be ‘grassroots’ movements arising from *everyday life* rather than being imposed from above. There is an apparent affinity between the concepts of *neo-tribe* and *subculture*, particularly as applied to youth (discussed below). Bennett (1999) argued that the concept of *neo-tribe* had become more appropriate for youth studies than *subcultur*e, given that the latter had been developed and shaped by critical theorists to exclusively represent forms of resistance to the structural features of capitalist society. Neo-tribe offered a wider range of theoretical possibilities.

Michel de Certeau’s work, in *The Practice of Everyday Life* (1980), was empirically-based, arising from a specific project funded by the French government to explore the concept of culture. A key feature of his analytical framework was the focus on the plurality of the *relationships between* individuals rather than on individuals themselves as actors. The meta-framework comprised: society’s *representations* (e.g., broadcasting a television programme); the user/consumer’s related *behaviour* (e.g., time spent watching television); and ‘what the cultural consumer “makes” or “does” during this time and with these images’ (p.xii). The main focus of the research was on this third component which was seen not as passive *consumption*, but as something more active, namely, a form of *production* (p.xiv). Individuals had agency in the form of *evasive* actions (‘procedures of everyday creativity’) or *tactics* deployed in everyday life (p.xiv), which was characterised by ‘its ruses, its fragmentation (the result of circumstances), its poaching, its clandestine nature, its tireless but quiet activity, in short, by its quasi-invisibility’ (p.31).^2^ However, de Certeau failed to spell out the overall motivation for such resistance (what is being resisted and why) and its relationship with macro-level processes of social change (Pink, 2012, p.18–19; Mitchell 2007). He seemed to consider the micro-sociology of everyday life as a work-in-progress which would eventually play a role in the macro-sociology of society, noting the parallels with Foucault’s achievement in identifying the neglected ‘microphysics of power’ and elevating it to a recognisable and significant feature of the social system (pp.45–49) and also with Bourdieu’s early anthropological research (pp.50–60).

The second volume of de Certeau’s project includes two empirical studies, one of various everyday features of physical/urban neighbourhoods and one of cooking (De Certeau et al., 1990/1998). However, while they demonstrate the creativity, richness and deep-rootedness of French everyday culture, they do not make its posited *resistant* characteristic explicit. The case studies in Highmore’s (2011) *Ordinary Lives* provide similar illustrations in an Anglo-Celtic context.

#### Cultural Studies

The British study of leisure was heavily influenced in the 1970s and 1980s by the work of the Centre for Contemporary Cultural Studies of Birmingham University, which was focussed primarily on working class youth groups seen as members of subcultures. Analysis of the music tastes, styles of dress and values of these groups concluded that they expressed resistance to structural, hegemonic forces of capitalism. Micro-level subcultural analysis was therefore articulated with traditional macro-level neo-Marxist class analysis. Five examples of the treatment of the everyday by cultural studies sociologists, are discussed below in chronological order.

In *The Devil Makes Work: Leisure in Capitalist Britain*, by John Clarke and Chas Critcher (1985), presented a seminal neo-Marxist macro-theoretical perspective on leisure (pp.40–41), set in the context of a British society which reflected the ‘social divisions and systematic inequalities inherent in the organisation of contemporary capitalism’ (p.178). The supposed freedoms offered by the market of leisure goods and services were presented as illusory, being the means by which the forces of capitalism bound people to the system as workers and consumers. Although not a major feature of the book, considerable space was devoted to discussing everyday life, in the form of ‘family life’ and the holiday (pp.164–170), which offered individuals relief from ‘two of the constraints of everyday life’, namely *time* and *place* (p.171). Despite these constraints, there was no significant level of political dissatisfaction among the population since:

most people’s lives are not motivated by political abstractions but by the concerns which secure and mark out a more intimate framework of home and family, friends and relatives. Impenetrable to the public influences of economic and political change, our private lives inform our sense of who we are. (Clarke & Critcher, 1985, p.233)

This exhibits a consciousness of the divide between the theoretically concerned researcher and the everyday lives of the researched, which reflects similar observations by Lefebvre, Maffesoli and Cohen and Taylor mentioned above and is a recurring theme in the other cultural studies discussions examined below. Clarke and Criticher were, however, anchored to the real world of the British socialism movement of the time (p.234), which was faced with a challenge since, to most people, ‘basic socialist ideas remain abstract, with no apparent connection to everyday life’ (p.233). Nevertheless, one way to make such a connection was via leisure, so the British political left was urged into political battle with leisure as a potent part of the ‘agenda of contemporary socialism’ (p.240).

Paul Willis’s (1990) study of young people in 1980s Britain, *Common Culture: Symbolic Work at Play in the Everyday Cultures of the Young*, introduced the concept of *necessary symbolic work*, which everyone engaged in on an everyday basis. This comprised the use of: language in communication; the active body as a ‘practice and symbolic resource’; drama, in the sense of ‘communicative interaction with others’ (cf. Goffman); and a combination of all of the above to engage in symbolic creativity or the ‘production of *new* meanings intrinsically attached to feeling, to energy, to excitement and psychic movement’ (p.285). This symbolic work had three outputs: *individual identities*; the *placing of individual identities in larger wholes*, such as race, class, gender, age or regional groups (or, in contemporary terms, possible ‘tribes’); and *development and affirmation of individuals’ own sense of vital capacities* and ‘how they might be applied to the cultural world’ (p.285). This took place in all modes of everyday activity, including paid and unpaid work, family and leisure, with the ‘main cultural materials and resources’ used in leisure being ‘cultural commodities’ (p.286). While some of these resources came from the market, consumption, for the young people Willis studied, was to be understood as ‘an active, not a passive, process’ (p.287). The pessimism of much postmodernist commentary on consumer society was rejected as simplistic, since the sheer quantity and variety of consumer products and the creativity of consumers had released ‘a profane explosion of everyday symbolic life and activity’. The ‘genie of common culture’ was ‘out of the bottle – let out by commercial carelessness’ (p.291).

John Fiske (1992) was very much concerned with the theme of the distance between the researchers and the researched, observing that ‘both academics in cultural and media studies and left-wing political theorists and activists have found the everyday culture of the people in capitalist societies particularly difficult to study either empirically or theoretically’ (p.154). The researchers were typically among the ‘socially advantaged and empowered’, while the researched were members of ‘subordinated social formations’ living in ‘conditions of oppression’ (p.154). Fiske nevertheless referred to examples of studies which had overcome these barriers, further suggesting that this could be achieved by researchers from a variety of backgrounds bringing to bear their ‘personal experience of living and practicing culture’ (p.159). In the intervening 30 years, this has surely been widely demonstrated in social researcher related to class, race and gender.

In regard to substantive theory, Fiske (1992) begins with Foucault’s contention that ‘society works on a highly elaborated system of surveying, and recording, ranking and individuating our everyday behaviours’ (p.161). Individual differences are not innate but result from this top-down power system disciplining individuals to fit into increasingly complex social, economic and technical roles. However, while Foucault (1980, p.95) argued that ‘points of resistance are present everywhere in the power network’, Fiske looks for the source of change in ‘social difference’, without which ‘there can be no social change’. So a ‘progressive theory of social difference needs to include, but must go beyond, the analysis of differences produced and controlled by the dominant social order’ (1992, p.163). As a source of social change he draws on Bourdieu and persons with different *habituses*, noting the ‘contradictory forces that make it difficult for some people to “settle” comfortably and make one habituated position their home’ (p.163). The process by which this results in social change is, however, not explored.

David Chaney, in *Cultural Change and Everyday Life* (2002), observes that social change, which has been a focus of the field of cultural studies, is being driven largely by what happens in everyday life. But tracking this process is a challenge because of the complexity and apparent, but not necessarily real, *irrationality* of everyday life compared with the *rationality* of the formal institutions of society (thus reflecting, although not citing, Habermas). The task is further exacerbated by culture being subject to a process of *fragmentation* as cultural forms proliferate and the boundaries between producers and consumers dissolve.

In *Reading the everyday*, Joe Moran (2005) addresses the neglect of the everyday in the British cultural studies tradition. He argues that the latter’s focus on youth culture, especially as expressed through symbolic and resistant practices, such as music, resulted in a neglect of the ‘boring, routine activities’ which constitute much of everyday life, for example, ‘waiting at bus stops’ (p.9).^3^ He observes that this may have been due to the perception that such activity seems to exist outside of history and social change (p.163), thereby lacking in macro-level significance. However, echoing Lefebvre’s comment on the role of the everyday life in defining a civilization, he argues that the everyday can be very much part of the macro-level historical process. As examples: the collapse of the eastern European communist regimes was the result not just of the denial of political freedoms but the ‘impoverishment of everyday life’ (p.164); the aim of terrorist organisations is often ‘to achieve the disruption and breakdown of everyday life by reducing people to a state of permanent anxiety’;^4^ and political leaders invoke the need to fight the forces of disruption in order to restore the pattern of ‘everyday life’, ‘ordinary life’ and ‘way of life’ (pp.166–167). Referencing Lefebvre and reflecting the preoccupation with social change, Moran concludes:

If we want to begin to transform our everyday lives for the better, perhaps we need to consider more closely how we think, talk about and represent them: to see the everyday not as the eternally tedious or bathetically comic residue of contemporary life, or simply as a sphere of overlooked ordinariness, but as the real space in which we lead our actual lives (Moran, 2005, p.169).

In contrast to Moran’s contention that cultural studies has neglected the everyday, Roberts (2006a, b) claims that the everyday has ‘become the common currency of much contemporary discourse on art and popular culture and cultural studies’ (p.1). However, as noted above, he argues that the typical cultural studies treatment has been limited historically and theoretically, given the absence of any ‘systematic Lefebvrian or Marxist engagement of the everyday in Anglo-American cultural studies before the rise of de Certeau’s influence’ (p.3). He seeks to remedy this by discussion of three historical periods: first, the period following the 1917 Russian Revolution; second, the post-World War II era from 1945 to 1965; and third, the period from 1966 to 1974, including ‘the incendiary moment of May 1968’ (pp.6–7). His discussion of the first of these indicates the origins of Soviet Marxist consideration of the everyday with a shift from exclusively economic and political concerns to inclusion of the cultural, in particular the question of how the everyday lives of the workers were to be reshaped as they moved from a revolutionary to post-revolutionary/socialist existence, exemplified by Leon Trotsky’s (1923/1973) *Problems of Everyday Life*.

#### Summary and Critique

In summary of the above overview, the three Marxist theorists, Lefebvre, Heller and Smith, sought to move the source of potential revolutionary change from the industrial/labour milieu to the cultural milieu of everyday life. The post-Marxist theorists carry on the concern with social change but with a further distancing from classic Marxist doctrine. The potential for bringing about desirable social change, or resisting undesirable change, lies in the everyday, but is differently contextualised. For Habermas, it lies in the resistance of the lifeworld to the predations of the media-steered systems, while for de Certeau, resistance arises spontaneously from everyday practice. In the case of Cohen and Taylor, the strictures of the everyday, or paramount reality, do not give rise to resistance but to efforts to escape, with an admission that this is unlikely to bring about social change. For Bourdieu and Maffesoli, rather than arising from the mass, social dynamism arises from the actions and interactions of groups, respectively class-fractions and neo-tribes. The cultural studies theorists continue the theme of social change arising from a culturally-based process located in the context of everyday life, but emphasise the challenge of actually studying such a phenomenon empirically.

The above body of work has attracted little criticism, an exception being Stephen Crook (1998), who questions two ‘related and questionable theses’ in the sociology of everyday life. First, he questions the proposition that individuals being born into an existing everyday lifeworld in which language and expected social behaviour patterns are ‘taken-for-granted’ is unique to everyday life. He argues that it occurs in individuals’ non-everyday experiences such as medical examinations and court hearings. Furthermore, taken-for-granted ‘social behaviour patterns’ are not homogeneous across everyday activity generally, but are likely to vary in complex ways in, for example, ‘working, playing, daydreaming, storytelling, joking, fighting and suffering pain’ (p.528). Second, the assumption that the contemporary everyday world is a source of authentic ‘living history’ with a direct link to the ‘pre-modern social totality’ is questioned (p.524), given two centuries of industrialisation and associated cultural change.

### Leisure and the Everyday

It is clear that many, possibly most, leisure scholars have embraced the micro-sociological style of research in recent decades, focussing on what Roberts (2011) refers to as ‘little leisures’. This involves studying participation in particular types of leisure (e.g., sport, tourism) and particular socio-demographic groups (e.g., women, the aged, youth). While such studies have often involved the everyday, this has not been an explicit focus; the concern has generally been the relative constraints on leisure experienced by the group. In some cases this is set in the context of some explicit macro-theoretical framework, but often the implied context is a concern for social justice. While it would be possible to explore this work in more detail, in this paper the focus is on research where the connection with the macro-level of theory is explicit. These are discussed in three groups: critical leisure theory; gender; and tourism. This is followed by two empirically-oriented examples.

#### Critical Leisure Theory

Chris Rojek’s (1995) *Decentring Leisure* is often credited with introducing postmodernism to the study of leisure, but his discussion of ‘everyday life’ in this volume (pp.105–108) was presented as part of his analysis of modernity. He drew initially on Lefebvre, but only for definitional purposes. He then referred to de Certeau and Fiske whose work he found unsatisfactory because their micro-level of analysis ignored such issues as class, gender, race and history. He was more approving of Cohen and Taylor’s approach, as discussed above, but argued that their equivocal conclusions implied that ‘the meaning of contemporary everyday life and leisure remains frustratingly elusive’ (p.108).^5^ In a part of the book examining ‘postmodern leisure’ he briefly discussed Maffesoli and ‘neo-tribalism’, noting that it was ‘an ordinary, unexceptional feature of daily life’ (pp.151–152).

Tony Blackshaw (2003), in *Leisure Life: Myth, Masculinity and Modernity*, examines the ‘mundane and the spectacular of the leisure life-world’ of ‘the lads’, a group of British working class young men. This is a rare example of a leisure studies research project which not only deals with the everyday, but references relevant theory, notably Maffesoli, de Certeau and the Situationist Debord. The ‘lads’ are presented as an example of Maffesoli’s concept of a ‘neo-tribe’, with their ‘leisure life-world’ based on a shared life history and mutuality. It is full of everyday rituals, such as frequent socializing and drinking in the same suburban pub, but with the routine punctuated by occasional highlights in the form of more extended nights of drinking, dancing and sexual pursuits in city-centre nightclubs. All this is intensely meaningful to ‘the lads’. However, as a study of everyday life, the account, while rich in detail, is somewhat one-dimensional in focusing on *leisure* life-worlds of the ‘lads’, largely ignoring relationships with other aspects of everyday life.

Blackshaw notes the gap between critical social theory and the conception of the everyday lives of the masses which had motivated the pioneers of the sociology of everyday life, observing: ‘the everyday leisure worlds of men and women – their inner and exterior lives and how these are individually experienced and shared with others – is one thing. Critical sociological discourse is quite another’ (2010, p.86). In particular, he argues, the focus of critical sociology of leisure on inequality in the context of class, gender and race is no longer appropriate. He attributes to Agnes Heller (discussed above), the idea that, from about the 1960s in Britain, there has been a ‘revolution of everyday life’, which has ‘challenged traditional conceptions of identity, youth, femininity and gender’ and has finally broken ‘the hitherto pervasive power of class’ (pp.86–87). However, his own replacement for the ‘traditional conceptions’ is not explicitly concerned with the everyday. Indeed, in a later work, he adopts a more exclusionary perspective on the ‘art of living’ in which leisure challenges individuals to ‘imagine another kind of life that feels like an escape from, not only their everyday one, but also from reason itself’ (Blackshaw, 2017, p.4).

#### Gender

Rita Felski (2000, p.30) observes that feminism has ‘traditionally conceived itself as a politics of everyday life’. In feminist leisure studies, it is common to find references to everyday life. For example, Green, et al. (1990, p.118), in a section of their book titled ‘Social control and everyday life’, noted that, while ‘beliefs and norms relating to women’s behaviour’ are embodied in ‘external forms, such as social policy, legislation and hierarchy’, they were also ‘internalised as part of the fabric of everyday life’. In the chapter on ‘Women’s leisure today’, they argued that women’s leisure should be studied as ‘part and parcel of the women’s lives as a whole’ (p.62). However, their own analysis of empirical data did not do this, relying instead on standard national activity-based survey data, and a presentation of their own data concerned exclusively with sport participation. Henderson et al. (1996, p.99) noted that, for many women, ‘their everyday lives tend to be holistic; work and leisure may coexist and be difficult to distinguish’. Wearing (1998, p.39) noted that feminist theorists had adopted a micro-sociological approach to ‘show how the meanings of leisure in the everyday lives of mainly middle-class white women can be different from their male counterparts’. Aitchison (2003, p.14) drew attention to the lack of research in areas of leisure such as ‘informal socialising, home-based leisure, the ‘everyday’ nature of leisure’. However, a surprising omission from this feminist leisure research agenda is the issue of work-life balance. Beginning with Hochschild’s (1997) *Time Bind*, the lead on this issue has been taken by non-leisure specialists (e.g., Boushey 2016; Negrey, 2012; Shippen, 2014; Schulte, 2014; Wajcman, 2015; Weeks, 2011).

#### Tourism

Tourism is often portrayed as the antithesis of everyday life. An example, as noted above, is Cohen and Taylor’s (1992) linking of holidays with the idea of escape from the everyday. Paralleling the tendency for leisure scholars to concentrate on the ‘higher forms’ of leisure, tourism scholars have tended to focus on the exotic, as exemplified by Urry’s (1990) popular concept of the ‘tourist gaze’. He observed that tourism experiences involved ‘some aspect or element which induces pleasurable experiences which are, by comparison with the everyday, out of the ordinary’ (p.11). He discussed overlaps between tourism and everyday activities, such as swimming, shopping and eating, but observed that, when such activities take place in tourism contexts, they tend to do so in unusual, non-everyday, environments, for example, museum re-creations of people’s domestic or working lives from the past (p.12). However, a number of tourism scholars have directly or indirectly critiqued the ‘gaze’ concept on various grounds and argued that tourism can become an everyday phenomenon. For example, less exotic forms of tourism include returning to the same caravan park year after year and meeting up with the same group of fellow-campers (Foley, 2017; Kyle & Chick, 2004) or going to resorts which offer a range of facilities which replicate the home environment, for example, the pubs and bingo provided for British holidaymakers in Spanish resorts. McCabe (2002) pointed out that the distinction between tourism and the everyday is modified by the fact that many everyday activities (such as eating, walking, drinking, conversation) are significant elements of the holiday, albeit experienced in different forms and environments and with a different tempo.

With increasing global mobility and cosmopolitanism, Franklin and Crang (2001, p.8) refer to the ‘tourism of everyday life’ and the ‘routinization of touristic sensibilities in everyday life’ (p.11), but do not expand on the idea. MacCannell (2001, p.25) questions Urry’s conception of touristic travel as ‘compensatory behaviour for a life that is … unpleasurable, flat and dull’. However, his main critique concerns the portrayal of the tourist gaze as a form of freedom when tourism is, in MacCannell’s view, invariably a controlled and contrived experience. He posits a ‘second gaze’, in which the potential tourist knowingly views the tourism experience being offered in advertising as deliberately over-selling the contrast with everyday life.^6^

Pons (2003) is also critical of Urry, seeking to ‘de-exoticise’ tourism by adopting a ‘being-in-the-world’ perspective on tourism:

The most relevant embodied practices through which we become tourists are everyday, ordinary, and often non-representational, practices. It is, therefore, insufficient in tourist studies to focus only on extraordinary practices, like sightseeing … Grasping only what is exceptional underplays both the continued relevance of the routines and habits in the configuration of tourist experience and the fact that the distinction between the everyday and the holiday is becoming increasingly fuzzy. (Pons, 2003, p.52)

Urry responded to a number of his critics in the second edition of his book (2002, pp.145–161), but his defence was concerned mostly with differing conceptualisations of the ‘gaze’, ignoring the type of everyday tourism where ‘gazing’ is not the point.

Larsen (2008) observes: ‘Discussion of everyday life is absent from tourism theory and research’ (p.22). Like Pons, he seeks to ‘de-exoticise’ tourism, noting that, due to modern home-based communications media, everyday spaces, far from being ‘grey and ordinary, … are full of exotic and spectacular signs’ (p.24). He also discusses one of the most popular, but neglected, forms of tourism, visiting friend and relatives, which, depending on distance and frequency, occupies a space between the everyday and the non-everyday.

#### Empirical

Two empirical contributions to the study of the phenomenon of everyday life are mentioned here: the Mass Observation project and the ‘leisure1-4’ taxonomy of time use.

The Mass Observation (MO) project was established in Britain in the 1930s, continued into the 1950s and was revived in the 1980s (Hubble, 2010). With theoretical links to surrealism and traditional ethnography, MO was an exploration of urban everyday life conducted by numerous paid and unpaid ‘observers’ (who observed public behaviour and conducted interviews), and ‘diarists’ (who wrote accounts of their own routine days). Teams were located in London and in Bolton in northern England. The project has been relatively neglected in overviews of everyday life research, with the exception of Highmore (2002b>, pp.75–112), who includes it as one among six significant contributions to the study of everyday life. The mainstream study of leisure has neglected MO, possibly due to criticisms of the project’s lack of representativeness, given the middle-class status of most observers/diarists (Pollen, 2013, pp.218 − 22) and to Kenneth Roberts’ critique of ‘mass society theory’ in his influential leisure sociology text (1978, pp. 41–61). There have, however, been exceptions, notably the work of Tomlinson and Tomlinson (1980), Snape (2016) and, using data from the revival phase, Wiseman (2021).

The ‘leisure1-4’ taxonomy of time use seeks to widen the scope of the study of leisure from the simple work-leisure binary to encompass eleven categories of time-use (Veal 2019b, pp.38–51): paid work; ‘grey work’ (travel to/from work, dealing with tele-messages in non-work time); education; domestic work; body maintenance; community activity; sleep; and four overlapping categories of leisure: (1) rest/recuperation from paid and unpaid work; (2) distraction/ entertainment; (3) family-orientated leisure (including extended family and close friendships); and (4) a residual ‘other’ category. Only the last of these encompasses the sorts of elevated forms of leisure which have preoccupied much leisure analysis, as mentioned at the beginning of the paper. The taxonomy provides a descriptive framework which offers potential for analysis of the everyday. However, it is limited by being a taxonomy only and functioning purely at the micro-level: the role of institutions – employment, family and community – being only implicit, while the role of commerce and related consumption activity is not identified. Incorporation of these elements could provide a more broadly-based framework for studying everyday leisure in its social and economic context.

## Conclusions

Leisure research has not entirely neglected the everyday, but it has been peripheral. In fact, as noted at the start of this paper, the theoretical emphasis has often been on the non-everyday, concentrating on relatively elevated and even élitist, concepts of leisure. Embracing the concept of everyday life can be seen as an antidote to this tendency. Roberts (2016) has stated that leisure researchers are ‘best advised to scan, take and use their selections from the stock of theories that have been built since the nineteenth century. This will best serve the study of … leisure: there is no need to build theories anew’ (p.188). The sociology of the everyday is part of the ‘stock of theories’ upon which the study of leisure should draw. This paper has filled a gap in the literature in exploring the links between the two fields of study. Further work will, however, be needed to realise the full potential of the everyday life perspective in the study of leisure. Two possibilities are highlighted here.

The first possibility arises from the proposition that a key role of social research is to address pressing issues of the time. As leisure was emerging as a field of study in the 1960 and 1970 s, the growth of leisure was itself an issue of the time (Roberts, 1978; Veal, 2019b). The corresponding contemporary issue is *work-life balance*. While the topic has been a focus of attention by a few leisure researchers (e.g., Roberts 2007; Veal, 2020), the leisure perspective has not had a high profile, considering the level of interest in popular discourse and particularly the work of feminist non-leisure specialist commentators, as noted above. These commentators have looked to everyday life as a key site of social change. Problems of work-life balance are a form of stressful change taking place in the experience of everyday life. The complexity of modern life and the diversity of contemporary lifestyles suggest that the traditional approaches to the study of leisure have little to offer this debate. Analyses involving an everyday life perspective, including everyday leisure, offer a basis for a significant contribution.

A second possibility is prompted by suggestions that research on leisure should ‘decentre’ leisure (Rojek, 1995) or treat it as more ‘inconsequential’ than hitherto (Roberts, 2011). Adopting an everyday life perspective would achieve this. The ‘leisure1-4’ taxonomy, possibly in extended form as discussed above, seeks to do this by making explicit the proposition that leisure is just one among many competing types of time use and that leisure activity itself comes in a variety of forms, playing a number of roles and interacting with a number of social and economic institutions.

If the study of leisure is to be relevant to the real world it should ideally reflect that world, which is one in which most leisure is everyday leisure.

## Data Availability

Not applicable.

## Footnotes

1 The ‘magic world of voices’ would today no doubt be widened to include social media.

2 As with Maffesoli and, indeed, all the theorists reviewed above, de Certeau was writing before the advent of social media, which clearly enhance the processes described.

3 Moran offers a quite detailed examination of the boring activity of waiting at bus stops. However, his book pre-dates the growth of mobile phone ownership and social media, which, arguably, have transformed the experience of waiting in queues for many.

4 More recently, in 2020–2021, a pandemic (COVID-19), rather than a terrorist organization, has similarly illustrated the significance of interruptions to everyday life.

5 While Rojek (1995) references the 1992 edition of Cohen and Taylor’s book, the pagination of his quotations and his ignoring of the significant ‘Introduction to the Second [1992] edition’ suggests that he was actually using the 1976 edition. This is ironic, given that the main change which Cohen and Taylor sought to address in the new introduction was the advent of postmodern theory – and that was Rojek’s main focus.

6 While MacCannell does not make this connection, it can be seen as paralleling De Certeau’s (1981, p.31) view of the individual’s sceptical approach to what is offered and portrayed in everyday life.
